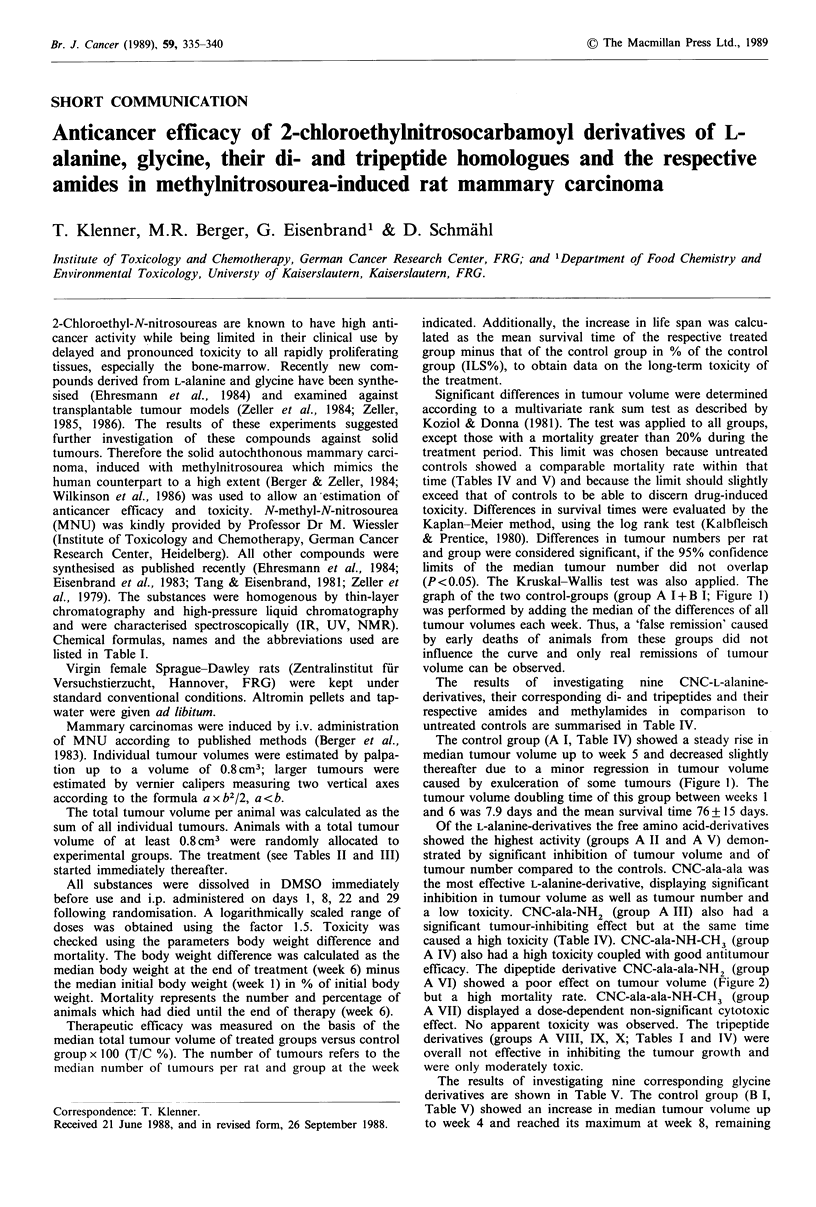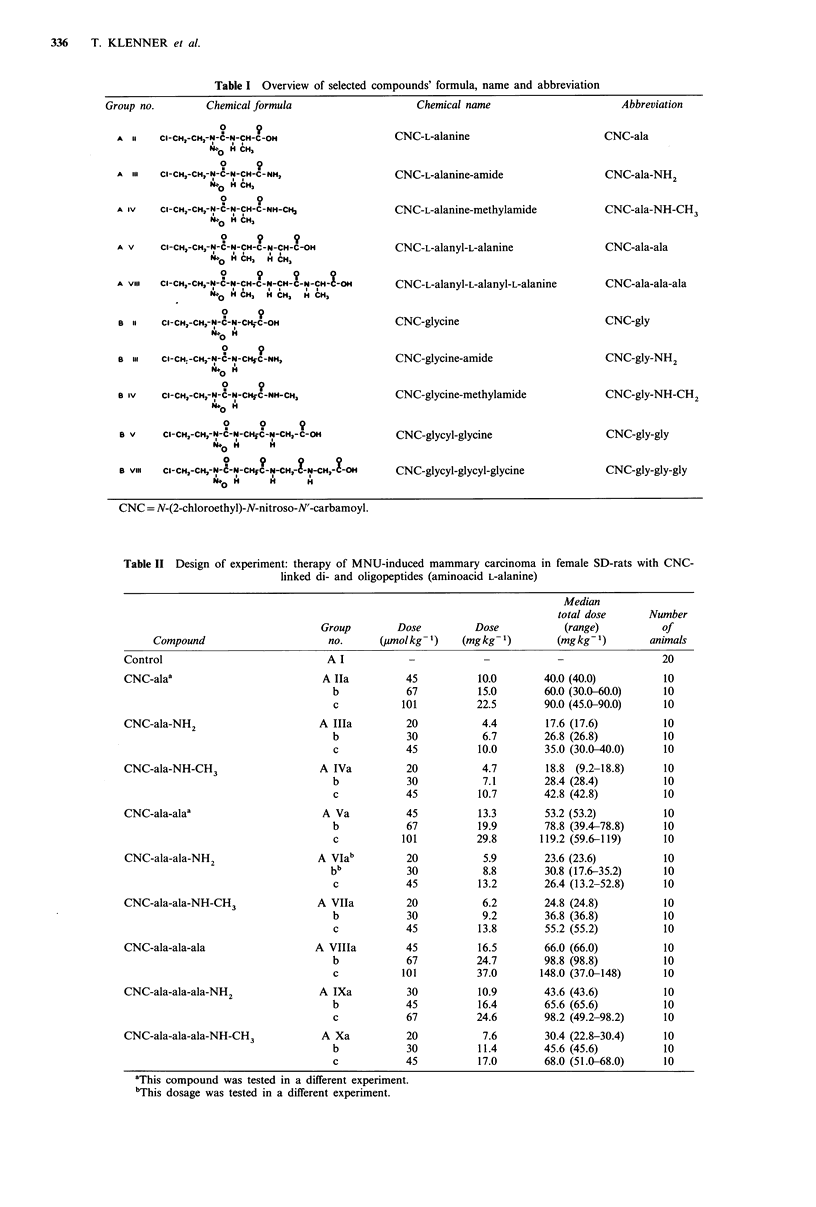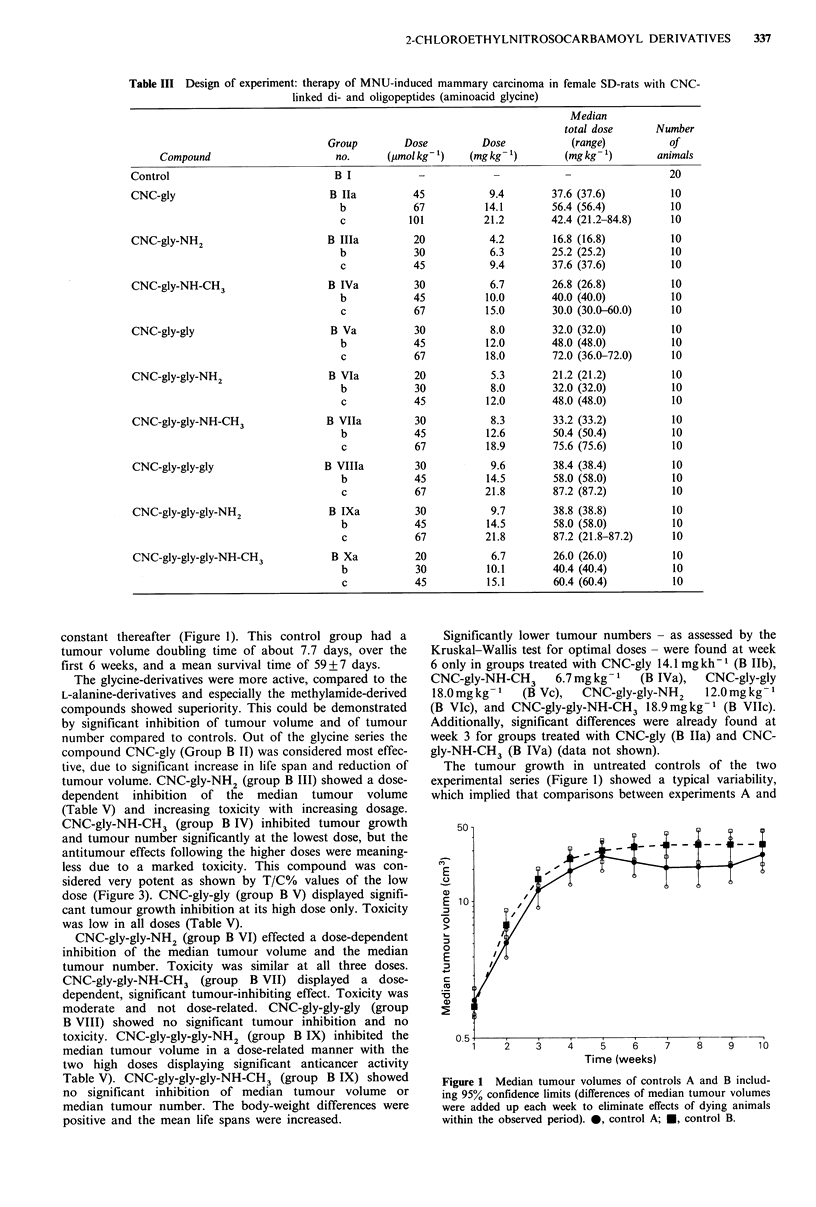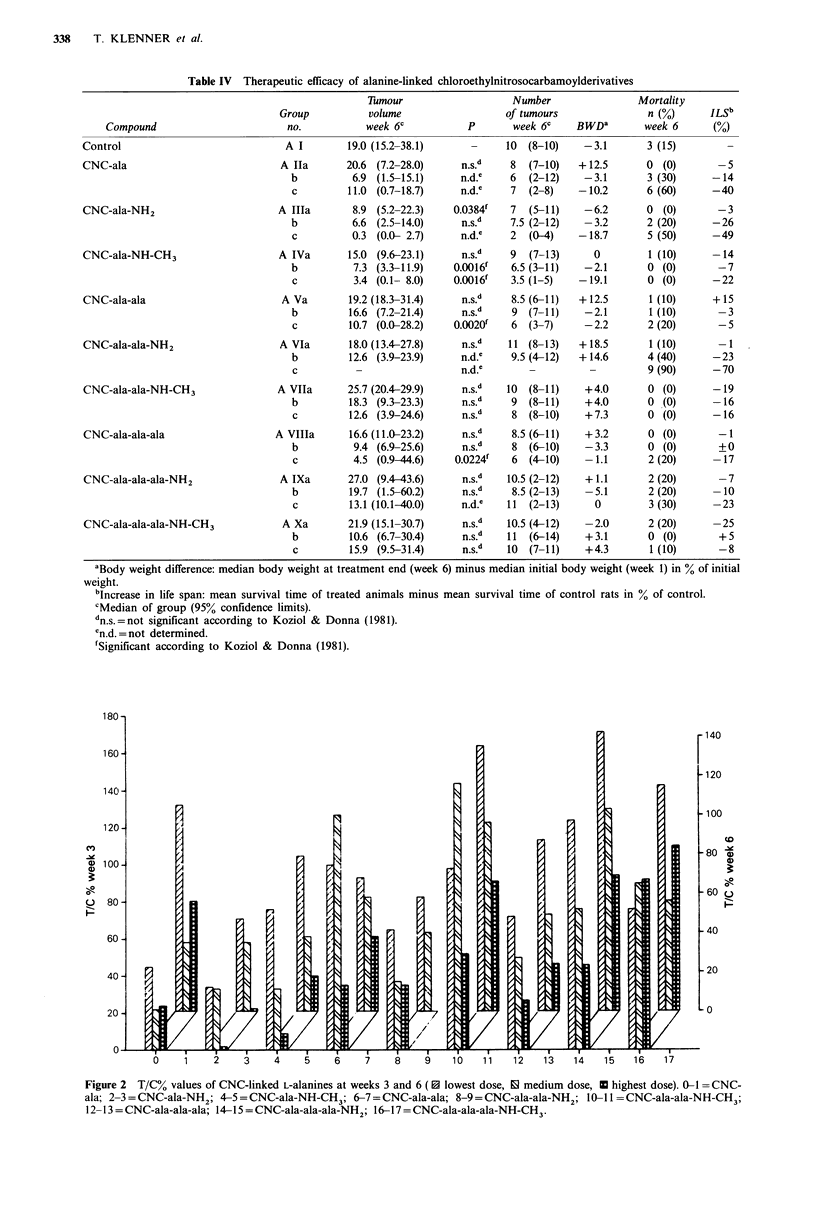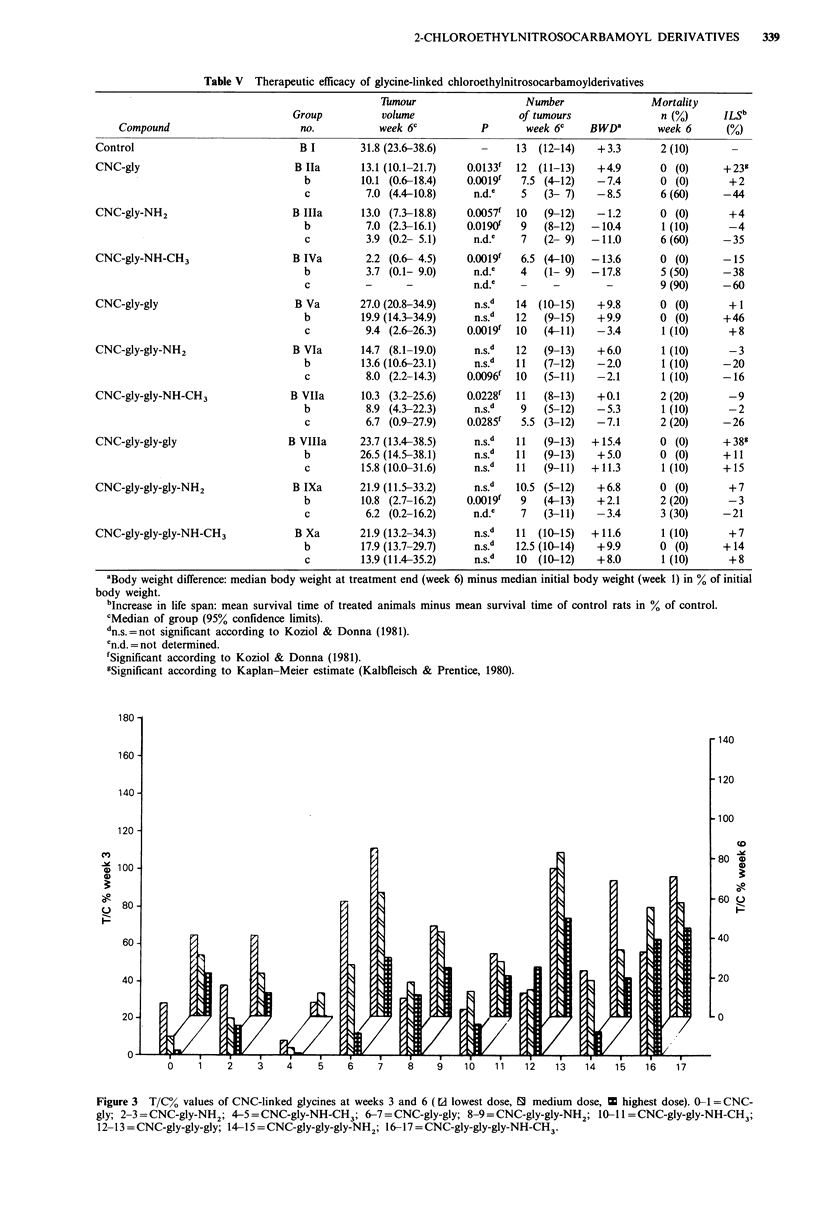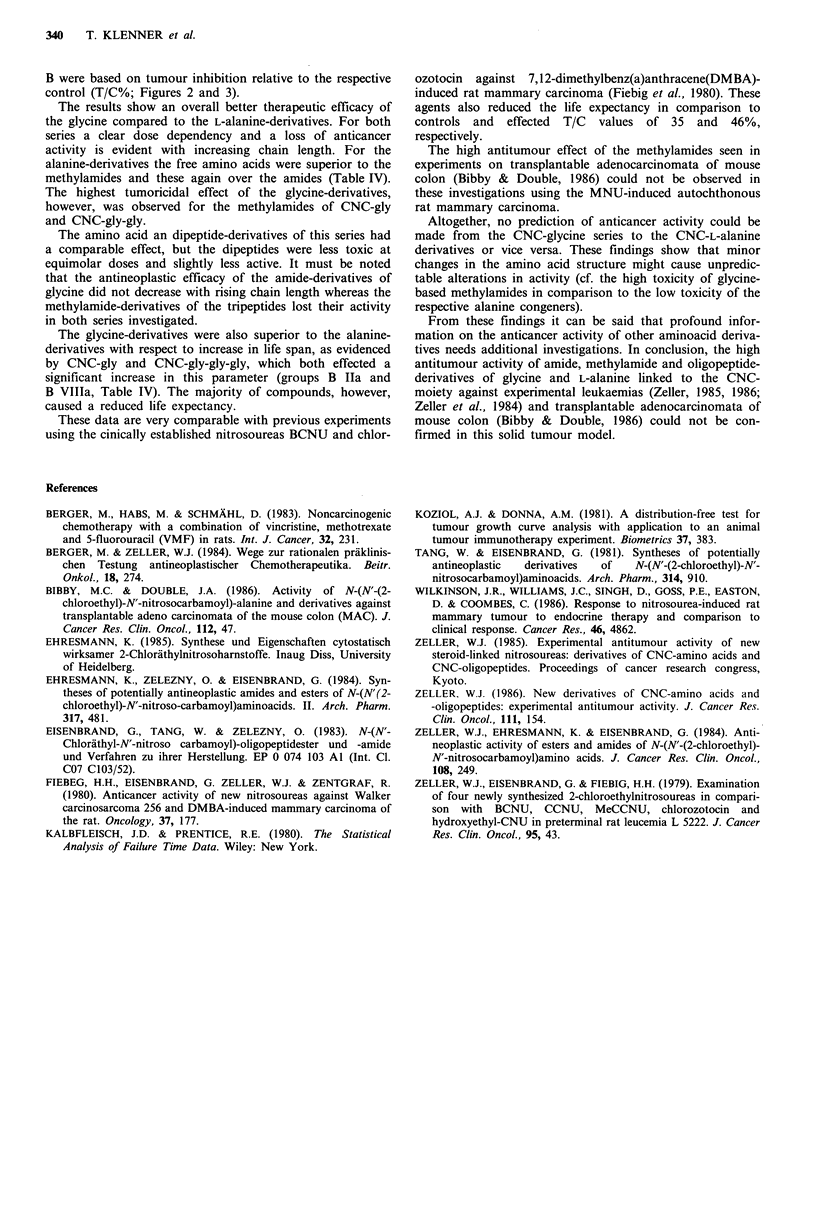# Anticancer efficacy of 2-chloroethylnitrosocarbamoyl derivatives of L-alanine, glycine, their di- and tripeptide homologues and the respective amides in methylnitrosourea-induced rat mammary carcinoma.

**DOI:** 10.1038/bjc.1989.66

**Published:** 1989-03

**Authors:** T. Klenner, M. R. Berger, G. Eisenbrand, D. SchmÃ¤hl

**Affiliations:** Institute of Toxicology and Chemotherapy, German Cancer Research Center, FRG.


*Br. J. Cancer* (1989), **59**, 335–340 © The Macmillan Press Ltd., 1989

SHORT COMMUNICATION

### Anticancer efficacy of 2-chloroethylnitrosocarbamoyl derivatives of L-alanine, glycine, their di- and tripeptide homologues and the respective amides in methylnitrosourea-induced rat mammary carcinoma

T. Klenner, M.R. Berger, G. Eisenbrand[1] & D. Schmähl

*Institute of Toxicology and Chemotherapy, German Cancer Research Center, FRG; and [1]Department of Food Chemistry and Environmental Toxicology, Universty of Kaiserslautern, Kaiserslautern, FRG.*

2-Chloroethyl-*N*-nitrosoureas are known to have high anticancer activity while being limited in their clinical use by delayed and pronounced toxicity to all rapidly proliferating tissues, especially the bone-marrow. Recently new compounds derived from L-alanine and glycine have been synthesised (Ehresmann *et al.*, 1984) and examined against transplantable tumour models (Zeller *et al.*, 1984; Zeller, 1985, 1986). The results of these experiments suggested further investigation of these compounds against solid tumours. Therefore the solid autochthonous mammary carcinoma, induced with methylnitrosourea which mimics the human counterpart to a high extent (Berger & Zeller, 1984; Wilkinson *et al.*, 1986) was used to allow an estimation of anticancer efficacy and toxicity. *N*-methyl-*N*-nitrosourea (MNU) was kindly provided by Professor Dr M. Wiessler (Institute of Toxicology and Chemotherapy, German Cancer Research Center, Heidelberg). All other compounds were synthesised as published recently (Ehresmann *et al.*, 1984; Eisenbrand *et al.*, 1983; Tang & Eisenbrand, 1981; Zeller *et al.*, 1979). The substances were homogenous by thin-layer chromatography and high-pressure liquid chromatography and were characterised spectroscopically (IR, UV, NMR). Chemical formulas, names and the abbreviations used are listed in Table I.

Virgin female Sprague–Dawley rats (Zentralinstitut für Versuchstierzucht, Hannover, FRG) were kept under standard conventional conditions. Altromin pellets and tap-water were given *ad libitum*.

Mammary carcinomas were induced by i.v. administration of MNU according to published methods (Berger *et al.*, 1983). Individual tumour volumes were estimated by palpation up to a volume of 0.8 $cm^3$; larger tumours were estimated by vernier calipers measuring two vertical axes according to the formula $a \times b^2/2$, $a<b$.

The total tumour volume per animal was calculated as the sum of all individual tumours. Animals with a total tumour volume of at least 0.8 $cm^3$ were randomly allocated to experimental groups. The treatment (see Tables II and III) started immediately thereafter.

All substances were dissolved in DMSO immediately before use and i.p. administered on days 1, 8, 22 and 29 following randomisation. A logarithmically scaled range of doses was obtained using the factor 1.5. Toxicity was checked using the parameters body weight difference and mortality. The body weight difference was calculated as the median body weight at the end of treatment (week 6) minus the median initial body weight (week 1) in % of initial body weight. Mortality represents the number and percentage of animals which had died until the end of therapy (week 6).

Therapeutic efficacy was measured on the basis of the median total tumour volume of treated groups versus control group × 100 (T/C %). The number of tumours refers to the median number of tumours per rat and group at the week indicated. Additionally, the increase in life span was calculated as the mean survival time of the respective treated group minus that of the control group in % of the control group (ILS%), to obtain data on the long-term toxicity of the treatment.

Significant differences in tumour volume were determined according to a multivariate rank sum test as described by Koziol & Donna (1981). The test was applied to all groups, except those with a mortality greater than 20% during the treatment period. This limit was chosen because untreated controls showed a comparable mortality rate within that time (Tables IV and V) and because the limit should slightly exceed that of controls to be able to discern drug-induced toxicity. Differences in survival times were evaluated by the Kaplan–Meier method, using the log rank test (Kalbfleisch & Prentice, 1980). Differences in tumour numbers per rat and group were considered significant, if the 95% confidence limits of the median tumour number did not overlap ($P<0.05$). The Kruskal–Wallis test was also applied. The graph of the two control-groups (group A I+B I; Figure 1) was performed by adding the median of the differences of all tumour volumes each week. Thus, a 'false remission' caused by early deaths of animals from these groups did not influence the curve and only real remissions of tumour volume can be observed.

The results of investigating nine CNC-L-alanine-derivatives, their corresponding di- and tripeptides and their respective amides and methylamides in comparison to untreated controls are summarised in Table IV.

The control group (A I, Table IV) showed a steady rise in median tumour volume up to week 5 and decreased slightly thereafter due to a minor regression in tumour volume caused by exulceration of some tumours (Figure 1). The tumour volume doubling time of this group between weeks 1 and 6 was 7.9 days and the mean survival time 76 ± 15 days.

Of the L-alanine-derivatives the free amino acid-derivatives showed the highest activity (groups A II and A V) demonstrated by significant inhibition of tumour volume and of tumour number compared to the controls. CNC-ala-ala was the most effective L-alanine-derivative, displaying significant inhibition in tumour volume as well as tumour number and a low toxicity. CNC-ala-$NH_2$ (group A III) also had a significant tumour-inhibiting effect but at the same time caused a high toxicity (Table IV). CNC-ala-NH-$CH_3$ (group A IV) also had a high toxicity coupled with good antitumour efficacy. The dipeptide derivative CNC-ala-ala-$NH_2$ (group A VI) showed a poor effect on tumour volume (Figure 2) but a high mortality rate. CNC-ala-ala-NH-$CH_3$ (group A VII) displayed a dose-dependent non-significant cytotoxic effect. No apparent toxicity was observed. The tripeptide derivatives (groups A VIII, IX, X; Tables I and IV) were overall not effective in inhibiting the tumour growth and were only moderately toxic.

The results of investigating nine corresponding glycine derivatives are shown in Table V. The control group (B I, Table V) showed an increase in median tumour volume up to week 4 and reached its maximum at week 8, remaining

Correspondence: T. Klenner.
Received 21 June 1988, and in revised form, 26 September 1988.

**Table I** Overview of selected compounds' formula, name and abbreviation

| *Group no.* | *Chemical formula* | *Chemical name* | *Abbreviation* |
|---|---|---|---|
| A II |  | CNC-L-alanine | CNC-ala |
| A III |  | CNC-L-alanine-amide | CNC-ala-$NH_2$ |
| A IV |  | CNC-L-alanine-methylamide | CNC-ala-NH-$CH_3$ |
| A V |  | CNC-L-alanyl-L-alanine | CNC-ala-ala |
| A VIII |  | CNC-L-alanyl-L-alanyl-L-alanine | CNC-ala-ala-ala |
| B II |  | CNC-glycine | CNC-gly |
| B III |  | CNC-glycine-amide | CNC-gly-$NH_2$ |
| B IV |  | CNC-glycine-methylamide | CNC-gly-NH-$CH_2$ |
| B V |  | CNC-glycyl-glycine | CNC-gly-gly |
| B VIII |  | CNC-glycyl-glycyl-glycine | CNC-gly-gly-gly |

CNC = *N*-(2-chloroethyl)-*N*-nitroso-*N'*-carbamoyl.

**Table II** Design of experiment: therapy of MNU-induced mammary carcinoma in female SD-rats with CNC-linked di- and oligopeptides (aminoacid L-alanine)

| *Compound* | *Group no.* | *Dose* ($\mu mol\,kg^{-1}$) | *Dose* ($mg\,kg^{-1}$) | *Median total dose (range)* ($mg\,kg^{-1}$) | *Number of animals* |
|---|---|---|---|---|---|
| Control | A I | – | – | – | 20 |
| CNC-ala[a] | A IIa | 45 | 10.0 | 40.0 (40.0) | 10 |
| | b | 67 | 15.0 | 60.0 (30.0–60.0) | 10 |
| | c | 101 | 22.5 | 90.0 (45.0–90.0) | 10 |
| CNC-ala-$NH_2$ | A IIIa | 20 | 4.4 | 17.6 (17.6) | 10 |
| | b | 30 | 6.7 | 26.8 (26.8) | 10 |
| | c | 45 | 10.0 | 35.0 (30.0–40.0) | 10 |
| CNC-ala-NH-$CH_3$ | A IVa | 20 | 4.7 | 18.8 (9.2–18.8) | 10 |
| | b | 30 | 7.1 | 28.4 (28.4) | 10 |
| | c | 45 | 10.7 | 42.8 (42.8) | 10 |
| CNC-ala-ala[a] | A Va | 45 | 13.3 | 53.2 (53.2) | 10 |
| | b | 67 | 19.9 | 78.8 (39.4–78.8) | 10 |
| | c | 101 | 29.8 | 119.2 (59.6–119) | 10 |
| CNC-ala-ala-$NH_2$ | A VIa[b] | 20 | 5.9 | 23.6 (23.6) | 10 |
| | b[b] | 30 | 8.8 | 30.8 (17.6–35.2) | 10 |
| | c | 45 | 13.2 | 26.4 (13.2–52.8) | 10 |
| CNC-ala-ala-NH-$CH_3$ | A VIIa | 20 | 6.2 | 24.8 (24.8) | 10 |
| | b | 30 | 9.2 | 36.8 (36.8) | 10 |
| | c | 45 | 13.8 | 55.2 (55.2) | 10 |
| CNC-ala-ala-ala | A VIIIa | 45 | 16.5 | 66.0 (66.0) | 10 |
| | b | 67 | 24.7 | 98.8 (98.8) | 10 |
| | c | 101 | 37.0 | 148.0 (37.0–148) | 10 |
| CNC-ala-ala-ala-$NH_2$ | A IXa | 30 | 10.9 | 43.6 (43.6) | 10 |
| | b | 45 | 16.4 | 65.6 (65.6) | 10 |
| | c | 67 | 24.6 | 98.2 (49.2–98.2) | 10 |
| CNC-ala-ala-ala-NH-$CH_3$ | A Xa | 20 | 7.6 | 30.4 (22.8–30.4) | 10 |
| | b | 30 | 11.4 | 45.6 (45.6) | 10 |
| | c | 45 | 17.0 | 68.0 (51.0–68.0) | 10 |

[a]This compound was tested in a different experiment.
[b]This dosage was tested in a different experiment.

**Table III** Design of experiment: therapy of MNU-induced mammary carcinoma in female SD-rats with CNC-linked di- and oligopeptides (aminoacid glycine)

| *Compound* | *Group no.* | *Dose* ($\mu mol\ kg^{-1}$) | *Dose* ($mg\ kg^{-1}$) | *Median total dose (range)* ($mg\ kg^{-1}$) | *Number of animals* |
|---|---|---|---|---|---|
| Control | B I | – | – | – | 20 |
| CNC-gly | B IIa | 45 | 9.4 | 37.6 (37.6) | 10 |
| | b | 67 | 14.1 | 56.4 (56.4) | 10 |
| | c | 101 | 21.2 | 42.4 (21.2–84.8) | 10 |
| CNC-gly-$NH_2$ | B IIIa | 20 | 4.2 | 16.8 (16.8) | 10 |
| | b | 30 | 6.3 | 25.2 (25.2) | 10 |
| | c | 45 | 9.4 | 37.6 (37.6) | 10 |
| CNC-gly-NH-$CH_3$ | B IVa | 30 | 6.7 | 26.8 (26.8) | 10 |
| | b | 45 | 10.0 | 40.0 (40.0) | 10 |
| | c | 67 | 15.0 | 30.0 (30.0–60.0) | 10 |
| CNC-gly-gly | B Va | 30 | 8.0 | 32.0 (32.0) | 10 |
| | b | 45 | 12.0 | 48.0 (48.0) | 10 |
| | c | 67 | 18.0 | 72.0 (36.0–72.0) | 10 |
| CNC-gly-gly-$NH_2$ | B VIa | 20 | 5.3 | 21.2 (21.2) | 10 |
| | b | 30 | 8.0 | 32.0 (32.0) | 10 |
| | c | 45 | 12.0 | 48.0 (48.0) | 10 |
| CNC-gly-gly-NH-$CH_3$ | B VIIa | 30 | 8.3 | 33.2 (33.2) | 10 |
| | b | 45 | 12.6 | 50.4 (50.4) | 10 |
| | c | 67 | 18.9 | 75.6 (75.6) | 10 |
| CNC-gly-gly-gly | B VIIIa | 30 | 9.6 | 38.4 (38.4) | 10 |
| | b | 45 | 14.5 | 58.0 (58.0) | 10 |
| | c | 67 | 21.8 | 87.2 (87.2) | 10 |
| CNC-gly-gly-gly-$NH_2$ | B IXa | 30 | 9.7 | 38.8 (38.8) | 10 |
| | b | 45 | 14.5 | 58.0 (58.0) | 10 |
| | c | 67 | 21.8 | 87.2 (21.8–87.2) | 10 |
| CNC-gly-gly-gly-NH-$CH_3$ | B Xa | 20 | 6.7 | 26.0 (26.0) | 10 |
| | b | 30 | 10.1 | 40.4 (40.4) | 10 |
| | c | 45 | 15.1 | 60.4 (60.4) | 10 |

constant thereafter (Figure 1). This control group had a tumour volume doubling time of about 7.7 days, over the first 6 weeks, and a mean survival time of $59 \pm 7$ days.

The glycine-derivatives were more active, compared to the L-alanine-derivatives and especially the methylamide-derived compounds showed superiority. This could be demonstrated by significant inhibition of tumour volume and of tumour number compared to controls. Out of the glycine series the compound CNC-gly (Group B II) was considered most effective, due to significant increase in life span and reduction of tumour volume. CNC-gly-$NH_2$ (group B III) showed a dose-dependent inhibition of the median tumour volume (Table V) and increasing toxicity with increasing dosage. CNC-gly-NH-$CH_3$ (group B IV) inhibited tumour growth and tumour number significantly at the lowest dose, but the antitumour effects following the higher doses were meaningless due to a marked toxicity. This compound was considered very potent as shown by T/C% values of the low dose (Figure 3). CNC-gly-gly (group B V) displayed significant tumour growth inhibition at its high dose only. Toxicity was low in all doses (Table V).

CNC-gly-gly-$NH_2$ (group B VI) effected a dose-dependent inhibition of the median tumour volume and the median tumour number. Toxicity was similar at all three doses. CNC-gly-gly-NH-$CH_3$ (group B VII) displayed a dose-dependent, significant tumour-inhibiting effect. Toxicity was moderate and not dose-related. CNC-gly-gly-gly (group B VIII) showed no significant tumour inhibition and no toxicity. CNC-gly-gly-gly-$NH_2$ (group B IX) inhibited the median tumour volume in a dose-related manner with the two high doses displaying significant anticancer activity Table V). CNC-gly-gly-gly-NH-$CH_3$ (group B IX) showed no significant inhibition of median tumour volume or median tumour number. The body-weight differences were positive and the mean life spans were increased.

Significantly lower tumour numbers – as assessed by the Kruskal–Wallis test for optimal doses – were found at week 6 only in groups treated with CNC-gly 14.1 mg $kh^{-1}$ (B IIb), CNC-gly-NH-$CH_3$ 6.7 mg $kg^{-1}$ (B IVa), CNC-gly-gly 18.0 mg $kg^{-1}$ (B Vc), CNC-gly-gly-$NH_2$ 12.0 mg $kg^{-1}$ (B VIc), and CNC-gly-gly-NH-$CH_3$ 18.9 mg $kg^{-1}$ (B VIIc). Additionally, significant differences were already found at week 3 for groups treated with CNC-gly (B IIa) and CNC-gly-NH-$CH_3$ (B IVa) (data not shown).

The tumour growth in untreated controls of the two experimental series (Figure 1) showed a typical variability, which implied that comparisons between experiments A and

**Figure 1** Median tumour volumes of controls A and B including 95% confidence limits (differences of median tumour volumes were added up each week to eliminate effects of dying animals within the observed period). ●, control A; ■, control B.

**Table IV** Therapeutic efficacy of alanine-linked chloroethylnitrosocarbamoylderivatives

| *Compound* | *Group no.* | *Tumour volume week 6*[c] | *P* | *Number of tumours week 6*[c] | *BWD*[a] | *Mortality n (%) week 6* | *ILS*[b] *(%)* |
|---|---|---|---|---|---|---|---|
| Control | A I | 19.0 (15.2–38.1) | – | 10 (8–10) | −3.1 | 3 (15) | – |
| CNC-ala | A IIa | 20.6 (7.2–28.0) | n.s.[d] | 8 (7–10) | +12.5 | 0 (0) | −5 |
| | b | 6.9 (1.5–15.1) | n.d.[e] | 6 (2–12) | −3.1 | 3 (30) | −14 |
| | c | 11.0 (0.7–18.7) | n.d.[e] | 7 (2–8) | −10.2 | 6 (60) | −40 |
| CNC-ala-$NH_2$ | A IIIa | 8.9 (5.2–22.3) | 0.0384[f] | 7 (5–11) | −6.2 | 0 (0) | −3 |
| | b | 6.6 (2.5–14.0) | n.s.[d] | 7.5 (2–12) | −3.2 | 2 (20) | −26 |
| | c | 0.3 (0.0– 2.7) | n.d.[e] | 2 (0–4) | −18.7 | 5 (50) | −49 |
| CNC-ala-NH-$CH_3$ | A IVa | 15.0 (9.6–23.1) | n.s.[d] | 9 (7–13) | 0 | 1 (10) | −14 |
| | b | 7.3 (3.3–11.9) | 0.0016[f] | 6.5 (3–11) | −2.1 | 0 (0) | −7 |
| | c | 3.4 (0.1– 8.0) | 0.0016[f] | 3.5 (1–5) | −19.1 | 0 (0) | −22 |
| CNC-ala-ala | A Va | 19.2 (18.3–31.4) | n.s.[d] | 8.5 (6–11) | +12.5 | 1 (10) | +15 |
| | b | 16.6 (7.2–21.4) | n.s.[d] | 9 (7–11) | −2.1 | 1 (10) | −3 |
| | c | 10.7 (0.0–28.2) | 0.0020[f] | 6 (3–7) | −2.2 | 2 (20) | −5 |
| CNC-ala-ala-$NH_2$ | A VIa | 18.0 (13.4–27.8) | n.s.[d] | 11 (8–13) | +18.5 | 1 (10) | −1 |
| | b | 12.6 (3.9–23.9) | n.d.[e] | 9.5 (4–12) | +14.6 | 4 (40) | −23 |
| | c | – | n.d.[e] | – | – | 9 (90) | −70 |
| CNC-ala-ala-NH-$CH_3$ | A VIIa | 25.7 (20.4–29.9) | n.s.[d] | 10 (8–11) | +4.0 | 0 (0) | −19 |
| | b | 18.3 (9.3–23.3) | n.s.[d] | 9 (8–11) | +4.0 | 0 (0) | −16 |
| | c | 12.6 (3.9–24.6) | n.s.[d] | 8 (8–10) | +7.3 | 0 (0) | −16 |
| CNC-ala-ala-ala | A VIIIa | 16.6 (11.0–23.2) | n.s.[d] | 8.5 (6–11) | +3.2 | 0 (0) | −1 |
| | b | 9.4 (6.9–25.6) | n.s.[d] | 8 (6–10) | −3.3 | 0 (0) | ±0 |
| | c | 4.5 (0.9–44.6) | 0.0224[f] | 6 (4–10) | −1.1 | 2 (20) | −17 |
| CNC-ala-ala-ala-$NH_2$ | A IXa | 27.0 (9.4–43.6) | n.s.[d] | 10.5 (2–12) | +1.1 | 2 (20) | −7 |
| | b | 19.7 (1.5–60.2) | n.s.[d] | 8.5 (2–13) | −5.1 | 2 (20) | −10 |
| | c | 13.1 (10.1–40.0) | n.d.[e] | 11 (2–13) | 0 | 3 (30) | −23 |
| CNC-ala-ala-ala-NH-$CH_3$ | A Xa | 21.9 (15.1–30.7) | n.s.[d] | 10.5 (4–12) | −2.0 | 2 (20) | −25 |
| | b | 10.6 (6.7–30.4) | n.s.[d] | 11 (6–14) | +3.1 | 0 (0) | +5 |
| | c | 15.9 (9.5–31.4) | n.s.[d] | 10 (7–11) | +4.3 | 1 (10) | −8 |

[a]Body weight difference: median body weight at treatment end (week 6) minus median initial body weight (week 1) in % of initial weight.
[b]Increase in life span: mean survival time of treated animals minus mean survival time of control rats in % of control.
[c]Median of group (95% confidence limits).
[d]n.s. = not significant according to Koziol & Donna (1981).
[e]n.d. = not determined.
[f]Significant according to Koziol & Donna (1981).

**Figure 2** T/C% values of CNC-linked L-alanines at weeks 3 and 6 (▨ lowest dose, ▧ medium dose, ▩ highest dose). 0–1 = CNC-ala; 2–3 = CNC-ala-$NH_2$; 4–5 = CNC-ala-NH-$CH_3$; 6–7 = CNC-ala-ala; 8–9 = CNC-ala-ala-$NH_2$; 10–11 = CNC-ala-ala-NH-$CH_3$; 12–13 = CNC-ala-ala-ala; 14–15 = CNC-ala-ala-ala-$NH_2$; 16–17 = CNC-ala-ala-ala-NH-$CH_3$.

**Table V** Therapeutic efficacy of glycine-linked chloroethylnitrosocarbamoylderivatives

| *Compound* | *Group no.* | *Tumour volume week 6*[c] | *P* | *Number of tumours week 6*[c] | *BWD*[a] | *Mortality n (%) week 6* | *ILS*[b] *(%)* |
|---|---|---|---|---|---|---|---|
| Control | B I | 31.8 (23.6–38.6) | – | 13 (12–14) | +3.3 | 2 (10) | – |
| CNC-gly | B IIa | 13.1 (10.1–21.7) | 0.0133[f] | 12 (11–13) | +4.9 | 0 (0) | +23[g] |
| | b | 10.1 (0.6–18.4) | 0.0019[f] | 7.5 (4–12) | −7.4 | 0 (0) | +2 |
| | c | 7.0 (4.4–10.8) | n.d.[e] | 5 (3– 7) | −8.5 | 6 (60) | −44 |
| CNC-gly-$NH_2$ | B IIIa | 13.0 (7.3–18.8) | 0.0057[f] | 10 (9–12) | −1.2 | 0 (0) | +4 |
| | b | 7.0 (2.3–16.1) | 0.0190[f] | 9 (8–12) | −10.4 | 1 (10) | −4 |
| | c | 3.9 (0.2– 5.1) | n.d.[e] | 7 (2– 9) | −11.0 | 6 (60) | −35 |
| CNC-gly-NH-$CH_3$ | B IVa | 2.2 (0.6– 4.5) | 0.0019[f] | 6.5 (4–10) | −13.6 | 0 (0) | −15 |
| | b | 3.7 (0.1– 9.0) | n.d.[e] | 4 (1– 9) | −17.8 | 5 (50) | −38 |
| | c | – – | n.d.[e] | – – | – | 9 (90) | −60 |
| CNC-gly-gly | B Va | 27.0 (20.8–34.9) | n.s.[d] | 14 (10–15) | +9.8 | 0 (0) | +1 |
| | b | 19.9 (14.3–34.9) | n.s.[d] | 12 (9–15) | +9.9 | 0 (0) | +46 |
| | c | 9.4 (2.6–26.3) | 0.0019[f] | 10 (4–11) | −3.4 | 1 (10) | +8 |
| CNC-gly-gly-$NH_2$ | B VIa | 14.7 (8.1–19.0) | n.s.[d] | 12 (9–13) | +6.0 | 1 (10) | −3 |
| | b | 13.6 (10.6–23.1) | n.s.[d] | 11 (7–12) | −2.0 | 1 (10) | −20 |
| | c | 8.0 (2.2–14.3) | 0.0096[f] | 10 (5–11) | −2.1 | 1 (10) | −16 |
| CNC-gly-gly-NH-$CH_3$ | B VIIa | 10.3 (3.2–25.6) | 0.0228[f] | 11 (8–13) | +0.1 | 2 (20) | −9 |
| | b | 8.9 (4.3–22.3) | n.s.[d] | 9 (5–12) | −5.3 | 1 (10) | −2 |
| | c | 6.7 (0.9–27.9) | 0.0285[f] | 5.5 (3–12) | −7.1 | 2 (20) | −26 |
| CNC-gly-gly-gly | B VIIIa | 23.7 (13.4–38.5) | n.s.[d] | 11 (9–13) | +15.4 | 0 (0) | +38[g] |
| | b | 26.5 (14.5–38.1) | n.s.[d] | 11 (9–13) | +5.0 | 0 (0) | +11 |
| | c | 15.8 (10.0–31.6) | n.s.[d] | 11 (9–11) | +11.3 | 1 (10) | +15 |
| CNC-gly-gly-gly-$NH_2$ | B IXa | 21.9 (11.5–33.2) | n.s.[d] | 10.5 (5–12) | +6.8 | 0 (0) | +7 |
| | b | 10.8 (2.7–16.2) | 0.0019[f] | 9 (4–13) | +2.1 | 2 (20) | −3 |
| | c | 6.2 (0.2–16.2) | n.d.[e] | 7 (3–11) | −3.4 | 3 (30) | −21 |
| CNC-gly-gly-gly-NH-$CH_3$ | B Xa | 21.9 (13.2–34.3) | n.s.[d] | 11 (10–15) | +11.6 | 1 (10) | +7 |
| | b | 17.9 (13.7–29.7) | n.s.[d] | 12.5 (10–14) | +9.9 | 0 (0) | +14 |
| | c | 13.9 (11.4–35.2) | n.s.[d] | 10 (10–12) | +8.0 | 1 (10) | +8 |

[a]Body weight difference: median body weight at treatment end (week 6) minus median initial body weight (week 1) in % of initial body weight.
[b]Increase in life span: mean survival time of treated animals minus mean survival time of control rats in % of control.
[c]Median of group (95% confidence limits).
[d]n.s. = not significant according to Koziol & Donna (1981).
[e]n.d. = not determined.
[f]Significant according to Koziol & Donna (1981).
[g]Significant according to Kaplan–Meier estimate (Kalbfleisch & Prentice, 1980).

**Figure 3** T/C% values of CNC-linked glycines at weeks 3 and 6 (▨ lowest dose, ▧ medium dose, ▦ highest dose). 0–1 = CNC-gly; 2–3 = CNC-gly-$NH_2$; 4–5 = CNC-gly-NH-$CH_3$; 6–7 = CNC-gly-gly; 8–9 = CNC-gly-gly-$NH_2$; 10–11 = CNC-gly-gly-NH-$CH_3$; 12–13 = CNC-gly-gly-gly; 14–15 = CNC-gly-gly-gly-$NH_2$; 16–17 = CNC-gly-gly-gly-NH-$CH_3$.

B were based on tumour inhibition relative to the respective control (T/C%; Figures 2 and 3).

The results show an overall better therapeutic efficacy of the glycine compared to the L-alanine-derivatives. For both series a clear dose dependency and a loss of anticancer activity is evident with increasing chain length. For the alanine-derivatives the free amino acids were superior to the methylamides and these again over the amides (Table IV). The highest tumoricidal effect of the glycine-derivatives, however, was observed for the methylamides of CNC-gly and CNC-gly-gly.

The amino acid an dipeptide-derivatives of this series had a comparable effect, but the dipeptides were less toxic at equimolar doses and slightly less active. It must be noted that the antineoplastic efficacy of the amide-derivatives of glycine did not decrease with rising chain length whereas the methylamide-derivatives of the tripeptides lost their activity in both series investigated.

The glycine-derivatives were also superior to the alanine-derivatives with respect to increase in life span, as evidenced by CNC-gly and CNC-gly-gly-gly, which both effected a significant increase in this parameter (groups B IIa and B VIIIa, Table IV). The majority of compounds, however, caused a reduced life expectancy.

These data are very comparable with previous experiments using the cinically established nitrosoureas BCNU and chlorozotocin against 7,12-dimethylbenz(a)anthracene(DMBA)-induced rat mammary carcinoma (Fiebig et al., 1980). These agents also reduced the life expectancy in comparison to controls and effected T/C values of 35 and 46%, respectively.

The high antitumour effect of the methylamides seen in experiments on transplantable adenocarcinomata of mouse colon (Bibby & Double, 1986) could not be observed in these investigations using the MNU-induced autochthonous rat mammary carcinoma.

Altogether, no prediction of anticancer activity could be made from the CNC-glycine series to the CNC-L-alanine derivatives or vice versa. These findings show that minor changes in the amino acid structure might cause unpredictable alterations in activity (cf. the high toxicity of glycine-based methylamides in comparison to the low toxicity of the respective alanine congeners).

From these findings it can be said that profound information on the anticancer activity of other aminoacid derivatives needs additional investigations. In conclusion, the high antitumour activity of amide, methylamide and oligopeptide-derivatives of glycine and L-alanine linked to the CNC-moiety against experimental leukaemias (Zeller, 1985, 1986; Zeller et al., 1984) and transplantable adenocarcinomata of mouse colon (Bibby & Double, 1986) could not be confirmed in this solid tumour model.

### References

BERGER, M., HABS, M. & SCHMÄHL, D. (1983). Noncarcinogenic chemotherapy with a combination of vincristine, methotrexate and 5-fluorouracil (VMF) in rats. *Int. J. Cancer*, **32**, 231.

BERGER, M. & ZELLER, W.J. (1984). Wege zur rationalen präklinischen Testung antineoplastischer Chemotherapeutika. *Beitr. Onkol.*, **18**, 274.

BIBBY, M.C. & DOUBLE, J.A. (1986). Activity of *N*-(*N'*-(2-chloroethyl)-*N'*-nitrosocarbamoyl)-alanine and derivatives against transplantable adeno carcinomata of the mouse colon (MAC). *J. Cancer Res. Clin. Oncol.*, **112**, 47.

EHRESMANN, K. (1985). Synthese und Eigenschaften cytostatisch wirksamer 2-Chloräthylnitrosoharnstoffe. Inaug Diss, University of Heidelberg.

EHRESMANN, K., ZELEZNY, O. & EISENBRAND, G. (1984). Syntheses of potentially antineoplastic amides and esters of *N*-(*N'(2*-chloroethyl)-*N'*-nitroso-carbamoyl)aminoacids. II. *Arch. Pharm.* **317**, 481.

EISENBRAND, G., TANG, W. & ZELEZNY, O. (1983). *N*-(*N'*-Chloräthyl-*N'*-nitroso carbamoyl)-oligopeptidester und -amide und Verfahren zu ihrer Herstellung. EP 0 074 103 A1 (Int. Cl. C07 C103/52).

FIEBEG, H.H., EISENBRAND, G. ZELLER, W.J. & ZENTGRAF, R. (1980). Anticancer activity of new nitrosoureas against Walker carcinosarcoma 256 and DMBA-induced mammary carcinoma of the rat. *Oncology*, **37**, 177.

KALBFLEISCH, J.D. & PRENTICE, R.E. (1980). *The Statistical Analysis of Failure Time Data*. Wiley: New York.

KOZIOL, A.J. & DONNA, A.M. (1981). A distribution-free test for tumour growth curve analysis with application to an animal tumour immunotherapy experiment. *Biometrics* **37**, 383.

TANG, W. & EISENBRAND, G. (1981). Syntheses of potentially antineoplastic derivatives of *N*-(*N'*-(2-chloroethyl)-*N'*-nitrosocarbamoyl)aminoacids. *Arch. Pharm.*, **314**, 910.

WILKINSON, J.R., WILLIAMS, J.C., SINGH, D., GOSS, P.E., EASTON, D. & COOMBES, C. (1986). Response to nitrosourea-induced rat mammary tumour to endocrine therapy and comparison to clinical response. *Cancer Res.*, **46**, 4862.

ZELLER, W.J. (1985). Experimental antitumour activity of new steroid-linked nitrosoureas: derivatives of CNC-amino acids and CNC-oligopeptides. Proceedings of cancer research congress, Kyoto.

ZELLER, W.J. (1986). New derivatives of CNC-amino acids and -oligopeptides: experimental antitumour activity. *J. Cancer Res. Clin. Oncol.*, **111**, 154.

ZELLER, W.J., EHRESMANN, K. & EISENBRAND, G. (1984). Antineoplastic activity of esters and amides of *N*-(*N'*-(2-chloroethyl)-*N'*-nitrosocarbamoyl)amino acids. *J. Cancer Res. Clin. Oncol.*, **108**, 249.

ZELLER, W.J., EISENBRAND, G. & FIEBIG, H.H. (1979). Examination of four newly synthesized 2-chloroethylnitrosoureas in comparison with BCNU, CCNU, MeCCNU, chlorozotocin and hydroxyethyl-CNU in preterminal rat leucemia L 5222. *J. Cancer Res. Clin. Oncol.*, **95**, 43.